# Potassium intake is associated with nutritional quality and actual diet cost: a study at formulating a low sodium high potassium (LSHP) healthy diet

**DOI:** 10.1017/jns.2021.104

**Published:** 2022-02-16

**Authors:** Farapti Farapti, Annas Buanasita, Dominikus R. Atmaka, Stefania W. Setyaningtyas, Merryana Adriani, Purwo S. Rejeki, Yoshio Yamaoka, Muhammad Miftahussurur

**Affiliations:** 1 Department of Nutrition, Faculty of Public Health, Universitas Airlangga, Surabaya 60115, Indonesia; 2 Post Graduate Doctoral Program, Faculty of Medicine, Universitas Airlangga, Surabaya 60132, Indonesia; 3 Nutrition Department, Surabaya Health Polytechnic, Surabaya 60282, Indonesia; 4 Department of Physiology, Faculty of Medicine, Universitas Airlangga, Surabaya 60132, Indonesia; 5 Department of Environmental and Preventive Medicine, Oita University Faculty of Medicine, Yufu 879-5593, Japan; 6 Department of Medicine, Gastroenterology and Hepatology Section, Baylor College of Medicine, Houston, TX, USA; 7 Gastroentero-Hepatology Division, Department of Internal Medicine, Faculty of Medicine-Dr. Soetomo Teaching Hospital, Universitas Airlangga, Surabaya 60286, Indonesia; 8 Institute of Tropical Disease, Universitas Airlangga, Surabaya 60115, Indonesia

**Keywords:** Diet cost, Healthy diet, Non-communicable disease, Nutritional assessment, Potassium, Sodium, DASH, dietary approaches to stop hypertension, LSHP, low sodium high potassium, NCD, non-communicable diseases

## Abstract

Increasing potassium and reducing sodium intake have been identified as a priority intervention to reduce non-communicable diseases. A low sodium high potassium (LSHP) healthy diet can be a predictor of overall dietary quality and is associated with higher diet costs. The present study was a randomised controlled-feeding trial, formulating menus of low sodium and potassium-rich healthy diet and comparing with usual diet (a control diet based on typical Indonesian diet) to assess the association of potassium intake in the menus with other nutritional contents and diet cost. Totally seventy menus, which consisted of LSHP diets and the usual diets for a 7-d cycle, were composed from the analysis of the Indonesian food composition database. The correlation coefficient of the potassium content of all menus with nutritional quality and diet cost was analysed using the Pearson test. Multiple linear regression analysis was performed to determine the most important nutrient in determining diet cost. A comparison of nutrition quality and diet cost from the two menus was analysed using the independent *t*-test. LSHP diet had significantly higher carbohydrate, protein, fibre, magnesium, calcium, vitamin C, potassium density and diet cost but lower total fat, saturated fat and energy density than the usual diet (*P* < 0⋅05). Furthermore, there was a strong positive correlation between fibre, potassium and diet cost (coefficient correlation of >0⋅8). Potassium is a nutrient that is closely related to diet quality although the cost of the diet often may inhibit its intake. A targeted and effective strategy is required to provide affordable food for achieving a sustainable nutrient-rich diet.

## Introduction

The global disease burden shows that non-communicable diseases (NCDs) are the leading cause of mortality and morbidity globally. There is increasing evidence that lifestyle and poor diet are the most important modifiable risk factors for NCDs^(1,2)^. Low potassium intake and high sodium intake have also been associated with a number of NCDs, including hypertension^(3)^, cardiovascular diseases^(4)^, obesity^(5)^, osteoporosis^(6)^, stroke^(7)^ and all-cause mortality^(8)^. In 2012, the World Health Organization (WHO) published guidelines regarding sodium and potassium intakes for adults and children and recommended the adoption of a healthy eating pattern to prevent the onset of diet-related diseases^(9,10)^.

As part of the salt reduction programme, WHO Member States agreed to reduce the global population's dietary salt intake by 30 % by 2025^(11,12)^. A recent systematic review reported that a total of seventy-five countries now have a national salt reduction strategy, twelve countries have reported reductions in population salt intake, nineteen reduced salt content in foods and six improvements in consumer knowledge, attitudes or behaviours relating to salt^(13)^. The successful decrease in sodium intake is based on reports that low sodium diet is likely to be feasible when considering such factors as cost, the need to meet other nutritional recommendations and having familiar meals to enhance acceptability^(14,15)^. In addition, the role of public health programme also contributed to increased awareness for reducing salt intake in the population^(16)^.

In contrast to the salt programme, programmes for increasing potassium intake at the population level are still rare and not progressive. However, it is suggested that a daily intake of 4700 mg of potassium intake has been identified as a priority intervention to reduce NCDs^(10,17,18)^. In reality, most populations around the world consume less than the recommended levels of potassium. The WHO suggests a potassium intake of at least 90 mmol/d (3510 mg/d) for adults; however, the WHO report revealed that potassium intake levels varied among studies and ranged from 45 to 100 mmol/d, with a median value of 73 mmol/d^(10)^. Other population studies showed wide ranges of potassium intake; some consumed approximately 35 mmol/d in the lowest group and 65 mmol/d in the highest group^(10,18)^. The newest meta-analysis showed potassium intake is less than half of the recommended intake and most people do not get enough potassium^(19)^.

Several randomised clinical studies and systematic reviews provided evidence and strengthened the conclusion that increasing potassium has beneficial effects on health. The intervention to increase potassium included supplementation and potassium source food; however, it is recommended that potassium should be consumed through food because of the safety and no upper limit^(10,20–22)^. The WHO and the Dietary Guidelines for Americans Advisory Committee recommendations for increased potassium consumption are found in many foods, including vegetables, fruits, beans and milk products^(10,20)^. The WHO also included potassium as a component of their healthy diet indicator (HDI)^(23)^. In addition, a previous study reported a potassium-rich diet or a potassium-dense diet was associated with higher diet costs^(15)^. This is relevant to many epidemiological studies that observed diets of better quality are costlier, healthy diets tend to be expensive and low-cost energy-dense diets also tend to be nutrient-poor^(24–26)^. However, most people who do not get enough potassium might indicate they consume low-quality diet, and this might lead to the global barrier to healthy eating particularly for low-income households^(19,20)^.

Many countries have applied dietary patterns and developed guidelines to maintain healthy eating in their population. Furthermore, the WHO has recommended the adoption of a healthy eating pattern to prevent the onset of diet-related diseases. These healthy diets, such as dietary approaches to stop hypertension (DASH) diet, Mediterranean diet and balanced diet, are not only focusing on sodium and potassium, but also essential nutrients categorised as nutrient-rich foods or nutrient-dense foods. These nutrients include potassium, magnesium, calcium, vitamin A (as carotenoids), vitamin C, vitamin E and fibre that are generally consumed in small amounts (less than 60 % of the recommended dietary allowance (RDA), especially for elderly people that appear to lack several nutrients in their diets^(27,28)^.

Since it is plausible that potassium intake can be a predictor of overall dietary quality and can lead to beneficial effects on human health, it is urgently needed to achieve the recommended potassium intake and nutrient-rich diet^(29–31)^. However, food cost affects how people eat healthy diets tend to be more expensive. This might be one reason why many people did not meet their nutrient needs, particularly the recommended potassium intake^(15)^. Therefore, the present study aimed to assess the association between potassium intake and other nutritional factors and diet cost to understand if potassium is a predictor of overall dietary quality and confirm that diet cost might be a barrier limiting many people from meeting the recommended potassium intake.

## Materials and methods

### Study design

The low sodium high potassium (LSHP) study was randomised, controlled-feeding trial comparing the effects of two dietary patterns on blood pressure and other metabolic markers among older people. The present study was ancillary to the LSHP diet trial which was conducted among adult participants in Surabaya, Indonesia. The two dietary patterns were tested in the present study which included the LSHP diet and the usual diet (control diet). We formulated the diet using a 7-d cycle menu, and the nutrient targets of two experimental diets used in the present study were similar to diets used in the DASH-sodium trial, the diet that recommended as a healthy dietary standard by several countries due to its nutritional and metabolic benefits^(20,32–35)^. The menu cycle consisted of three meals (breakfast, lunch and dinner) and two snacks (mid-morning and afternoon). So, totally two dietary patterns provided seventy menus, each LSHP diet and usual diet consisted of thirty-five menus. The menus were developed based on the traditional Indonesian menu, the foods most consumed by the Indonesian population and culturally acceptable.

### Food composition database compilation

Two dietitians with long-standing experience in nutritional fieldwork developed food compositions high in potassium and sodium. The food composition data for calculating nutrients were based on the two Indonesian food composition databases (Tabel Komposisi Pangan Indonesia/TKPI 2017 and Data Komposisi Bahan Makanan/DKBM 2017) (available at www.panganku.org) as the primary nutrient data sources^(36)^. A total of 1151 food items from TKPI and 1375 food items from DKBM were available for analysis. From the food list, we deleted similar food items, resulting in 1146 food items after sorting based on food names. Thereafter, we sorted the food list based on potassium content per 100 g and sodium content per 100 g. Then, we classified the food list differently for both potassium and sodium into four categories: (1) very high (≥1000 mg/100 g), (2) high (500–999 mg/100 g), (3) medium (100–499 mg/100 g) and (4) low (<100 mg/100 g). After classifying the food list, we ranked each category based on potassium and sodium content. Food lists that have higher potassium and sodium ratio were prioritised for use in the menu development.

### LSHP menu development

From the food database, we developed menus that fulfil the recommendation of high potassium and low sodium intakes. We called the LSHP diet. We formulated the diet using a 7-d cycle menu to develop the two experimental dietary patterns, the LSHP diet and the usual diet. All menus were developed at energy levels of 1800 kcal, potassium 4700 mg and sodium 1500 mg for the LSHP diet and potassium 2000 mg and sodium 2300 mg for the usual diet. We prepared the energy diet for the elderly age group based on the Indonesian RDA at approximately 1800 mg/d both for the LSHP diet and the usual diet^(37)^.

The nutritional composition of the foods or nutrient content of the diets was calculated using the Nutri survey software (version 2007). All foods used in the daily menu had no missing nutrient values in the database. All foods and ingredients were weighed on an electronic scale and balanced to the nearest 0⋅5 g, except for salt, oils and foods weighing less than 10 g which were weighed to the nearest 0⋅1 g. To ensure uniformity and consistency of nutrients, menus were developed with similar foods and recipes. For setting sodium content, we applied less salt to regular salted foods or recipes for the LSHP diet, and more salt was added to foods or recipes for the usual diet.

We prepared similar menus to the DASH diet. The diet is recommended as a healthy dietary standard and applied by several countries in the world. The DASH diet is rich in fruits and vegetables and limits the consumption of sugar, salt/sodium and total fat. It also provides increased amounts of potassium, calcium, magnesium and fibre. Furthermore, it parallels the healthy diet menus based on adherence to WHO's 2015 nutrition guidelines. The HDI assesses seven components which include ≥400 g of fruits and vegetables per day, total fat of <30 % for total energy, <10 % saturated fatty acid for total energy and ≥25 g/d dietary fibre^(23)^. Specifically, for potassium intake, we adjusted the 2019 Indonesian RDA to 4700 mg/d and sodium 1500 mg/d^(34)^.

### Usual diet

We compiled a control diet as a comparison to the LSHP diet which is propositional to energy intake. A control diet was made based on regular standards of what many Indonesians eat, and called it as usual diet. The usual menu has the same energy values as the LSHP menu. However, the main difference of those menus is potassium and sodium content: potassium 4700 mg and sodium 1500 mg for the LSHP diet and potassium 2000 mg and sodium 2300 mg for the usual diet. Similar to the LSHP diet, the menu cycle of the usual diet consisted of three meals and two snacks. In the applied menus, for example for snack, we chose cheese bread for the usual diet rather than fresh salad, juice and smoothies in the LSHP diet. In other cycle menus, two groups got the same menus such as juice; however, we chose lower potassium source foods from the fruits group for the usual diet.

### Nutritional quality

The assessment of nutritional quality was based on the mean of energy-dense diets (energy density), nutrient-dense foods (potassium density), the amount (g) of fruit and vegetable per day, the amount (g) of dietary fibre per day and nutrient content of macronutrient and micronutrient such as calcium, magnesium and vitamin C. All nutrition compositions were obtained from a 7-d cycle menu.

Dietary energy density (kcal/g) was calculated by dividing total dietary energy by the edible weight of foods. Potassium density is the potassium consumption per calorie intake (mg/kcal/d). In the present study, the association between the potassium content in the diet and nutritional quality was measured using the Pearson correlation. Furthermore, we used the independent *t*-test to compare the nutritional quality between the LSHP diet and the usual diet.

### Actual diet cost

Actual diet cost was the mean cost of the total 7-d cycle menu that was calculated per 1800 kcal of food purchased by using actual expenditures and a standard food price database. To calculate the cost of the diet, we collected regular prices from some major grocery retailers in Surabaya and food price databases. A standard food price database was obtained by estimating the standard price of each of the food items from the information centre of national strategic food price (PIHPS/hargapangan.id), the information system of availability and price development of food staples in East Java (siskaperbapo.com) and prices of food staples in Surabaya's traditional market. After adjustment for preparation and waste, food prices in the databases were expressed in rupiah (IDR) per 100 g of edible portion. All prices were collected between September and November 2019.

### Statistical analysis

Univariate analysis was performed in the table to show the classification of potassium food sources. The content of potassium from source foods (per 100 g) was divided into three categories in each food group, that is, >1000, 500–1000 and 100–500 mg based on two Indonesian food composition databases (Tabel Komposisi Pangan Indonesia/TKPI 2017 and Data Komposisi Bahan Makanan/DKBM 2017). Furthermore, the classification based on the food types was also presented.

Bivariate analysis was used to measure the correlation between the LSHP diet and diet quality indicators, in terms of nutrient value and actual diet cost. Statistical analysis was performed using the BMI SPSS software. The statistical analysis started by performing the Kolmogorov–Smirnov test to assess the normality of data distribution. Since all data were distributed normally, correlation analysis of potassium intake of the 7-d cycle menu and actual diet cost was performed using the Pearson correlation test and the difference of actual diet cost between the LSHP diet and the usual diet was assessed using the independent *t*-test. Linear regression analysis was performed to determine the correlation between diet cost as a dependent variable and other nutrients as independent variables. The result is considered statistically significant if *P* < 0⋅05.

## Results

The food list containing high potassium was identified through sorting of the food list based on potassium content per 100 g and classifying based on the food group source of carbohydrate, protein, fat, fruits, vegetables and dairy food. Supplementary Table S1 shows that skim milk and flour fortified with dairy were sources of dairy food containing potassium >1000 mg/100 g food weight. Plant protein from the bean group and some kinds of fish were categorised as high potassium food. Fruits and vegetables as sources of potassium generally contained potassium content of 100–1000 mg/100 g. Carbohydrates and proteins, on the other hand, had low potassium content.

The difference in the nutrient content between the LSHP diet and the usual diet can be seen in Table 1. It showed that carbohydrate, protein, fibre, magnesium, calcium, vitamin C and potassium density were significantly higher in the LSHP diet compared to the usual diet. On the other hand, the LSHP diet contained lower total fat, saturated fat and energy density than the usual diet. Furthermore, the diet cost of LSHP menus was more expensive than that of the usual diet.

**Table 1.** Macronutrient and micronutrient of the LSHP and usual diets

	High potassium diet (*n* 35 menus/7-d cycle)	Usual potassium diet (*n* 35 menus/7-d cycle)	*P* (significancy)
Energy	1836⋅27 ± 33⋅85	1811⋅14 ± 50⋅87	0·3
Carbohydrate	299⋅7 ± 17⋅34 (65⋅19 %)	276⋅63 ± 11⋅17 (61⋅13 %)	0·012*
Fat	46⋅54 ± 6⋅41 (22⋅73 %)	54⋅04 ± 6⋅01 (26⋅8 %)	0·043*
Protein	73⋅44 ± 4⋅65 (16 %)	66⋅77 ± 4⋅77 (14⋅92 %)	0·021*
Fibre (g)	30⋅87 ± 7⋅08	16⋅53 ± 3⋅01	<0·001*
Potassium	4577⋅74 ± 119⋅29	2127⋅11 ± 75⋅86	<0·001*
Sodium	1449⋅3 ± 136⋅77	2387⋅94 ± 172⋅26	<0·001*
Magnesium	291⋅33 ± 43⋅42	235⋅07 ± 29⋅79	0·015*
Calcium	840⋅17 ± 146⋅10	524⋅74 ± 83⋅4	<0·001*
Vitamin C	328⋅12 ± 266⋅75	97⋅14 ± 21⋅01	0·04*
Vitamin E	4⋅46 ± 3⋅11	6⋅01 ± 1⋅67	0·27
Vitamin A	2196⋅16 ± 2050⋅9	1613⋅24 ± 806⋅66	0·5
Saturated fat	5⋅86 ± 4⋅32	12⋅2 ± 3⋅34	0·01*
Cholesterol	235⋅89 ± 81⋅99	293⋅37 ± 70⋅61	0·185
Energy density	1⋅29 ± 0⋅89	2⋅02 ± 0⋅14	<0·001*
Potassium density	2⋅49 ± 0⋅99	1⋅18 ± 0⋅54	<0·001*
Fruits (g)	737⋅14 ± 115⋅10	150 ± 0⋅00	<0·001*
Vegetables (g)	223⋅57 ± 75⋅09	179⋅29 ± 24⋅91	0·18
Diet cost (IDR)	35 192.10 ± 3318⋅27	26 205.71 ± 3678⋅74	<0·001*

* Significant *P* < 0⋅05, independent *t*-test.

The Pearson test was used to assess the correlation between potassium content and other nutrients in all menus (Table 2). The result showed a positive relationship between potassium content and the nutrients in all menus: (1) fibre, calcium, fruits, potassium density and diet cost correlated strongly with potassium (coefficient correlation of >0⋅8); (2) potassium correlated moderately with carbohydrate, protein, magnesium, vitamin C, total fat and saturated fat and (3) a weak correlation was observed between energy and potassium. Further analysed by linear regression, the result of the study showed a significant correlation between diet cost and energy, carbohydrate, fibre, fruits, magnesium and potassium. Moreover, fibre and potassium were the most important nutrients in determining cost (Table 3).

**Table 2.** Correlation between potassium and other nutrients

	Total		LSHP diet		Usual diet	
	Coefficient correlation	*P* value	Coefficient correlation	*P* value	Coefficient correlation	*P* value
Energy (kcal/d)	0·279^a^	0·334*	−0·58	0·17	−0·001	0·99
Carbohydrate (g)	0·613	0·02*	−0·57	0·18	−0·66	0·11
Fat (g)	−0·518	0·06	0·65	0·11	0·07	0·88
Protein (g)	0·602	0·023*	0·29	0·53	0·28	0·54
Fibre (g)	0·787	0·001*	0·58	0·01*	0·06	0·39
Sodium (g)	0·201	0·49	−0·38	0·41	0·24	0·61
Magnesium (g)	0·62	0·018*	−0·37	0·41	0·19	0·69
Calcium (g)	0·832	<0·001*	0·29	0·54	0·5	0·25
Vitamin C (mg)	0·551	0·041*	0·07	0·88	−0·39	0·39
Vitamin E (mg)	−0·289	0·316	0·3	0·51	0·72	0·07
Vitamin A (μg)	0·229	0·43	0·64	0·12	−0·34	0·46
Saturated fat	−0·635	0·015*	0·42	0·35	0·6	0·15
Cholesterol	−0·38	0·18	−0·29	0·53	0·31	0·49
Energy density	−0·956	<0·001*	−0·18	0·7	0·34	0·45
Potassium density	0·998	<0·001*	0·92	0·003*	0·78	0·04*
Fruits (g)	0·975	<0·001*	0·58	0·18	0·61	0·15
Vegetables (g)	0·358	0·209	−0·52	0·23	−0·46	0·29
Diet cost (IDR)	0·809	<0·001*	0·45	0·31	0·68	0·09

LSHP, low sodium high potassium.

a All nutrition compositions were obtained from a 7-d cycle menu.

* Significant *P* < 0·05, Pearson's test.

**Table 3.** Linear regression analysis between diet cost and other nutrients

	Standadised coefficients (*β*)	*P* value
Energy (kcal/d)	0·46	0·007*
Carbohydrate (g)	−0·49	0·02*
Potassium	0·53	0·004
Magnesium	−0·48	0·023*
Fibre (g)	1·22	0·04*
Vegetables (g)	−0·39	0·013

* Significant *P* < 0·05.

## Discussion

To the best of our knowledge, this is the first study focusing on the formulation of daily menus with recommended potassium content and analyses of potassium content and actual diet cost in the menus. Population studies demonstrated that globally, most people of all ages consume less than the recommended potassium intake^(10)^. Since potassium intake was associated with metabolic syndrome and cardiovascular disease, it is important to fulfil the daily recommended potassium intake. The present study formulated a chronic disease-related diet, with a focus on LSHP foods that are palatable and mostly consumed by the Indonesian population. Furthermore, we adopted the DASH diet, a healthy dietary standard approved by several countries due to its benefit in terms of metabolic and nutritional status^(32–34)^.

Before formulating the daily menus of high recommended potassium healthy diet, it is foremost important to know the potassium food sources and their characteristics. Secondly, the obstacle to arranging the menus of a healthy diet should be identified. In the present study, food sources containing more than 500 mg potassium per 100 g were fruits, vegetables and plant protein, especially nut and dairy food. Conversely, most of the carbohydrate and fat food sources contained low potassium. Our result is similar to a previous study that reported that highly refined wheat flour contains less than half of the potassium level of complete flour and refined fats or sugars have very low potassium content and their presence or addition in foods results in a lowering of potassium concentration as well as nutritional density^(20,21,35)^. Finally, we chose foods that are low-cost sources of potassium such as potatoes, bananas and carrots to meet targeted dietary recommendations. This is similar to a previous study that highlighted that it is important to identify and promote lower-cost food as an effective approach to improve potassium intake^(15)^.

Potassium is an essential element and is found in all foods. However, previous studies demonstrated that food sources of potassium come from particularly fruits and vegetables^(20,30)^. Several large surveys have been published indicating that fruits and vegetables are the richest food sources of potassium among children^(38)^, adult^(39)^ and the elderly^(28)^. Many fresh fruits and vegetables are rich in potassium. In the present study, however, fruits had a strong correlation with potassium (*r* = 0⋅975, *P* = 0⋅000), but not vegetables. Dietary guidelines also suggested the consumption of adequate fruits and vegetables ranging from 400 to 600 mg/d^(40)^. Available empirical evidence have also found that fruits and vegetables are substantially more nutrient-dense with low energy content than other food groups, as evident by their highest nutrient density score^(24,25,27)^. Therefore, adequate intakes of fruits and vegetables, which are the major sources of potassium, are highly recommended. Besides the potassium benefit, fruit and vegetable intakes were associated with reduced risk of cardiovascular disease, cancer and all-cause mortality^(29–31)^. Unfortunately, despite these established benefits, most people consume less fruits and vegetables than recommended^(41–45)^.

The difficulty encountered in formulating the LSHP diet is related to energy content. The present study showed that the higher the potassium content in the menus serving, the higher the macronutrient intake (energy, protein and carbohydrate). It was particularly challenging to arrange the menus containing calorie restriction with high potassium and low sodium content, particularly for obesity treatment. Consistently, the previous studies showed that sodium and potassium intakes were positively correlated with energy intake^(46,47)^. The same trend was observed by a previous research which reported that potassium intake was correlated with total calories but further stated that potassium alone, which is not more effective than the DASH diet, improves endothelial function^(48)^. On the contrary, other studies confirmed that high potassium intake was associated with lower body mass index (BMI)^(30,49,50)^. The increase in dietary potassium consumption was a strong predictor of a decline in BMI, which might have been caused by the potassium density^(49)^. The study showed that participants who achieved a higher loss in BMI also had higher potassium density. The present study revealed that potassium intake had a strong positive correlation with potassium density, while it had a negative correlation with energy density. The present findings also revealed that potassium density was significantly higher in the LSHP diet compared to the usual diet. Thus, it is likely that potassium density, defined as potassium consumption per calorie intake, might become a useful marker to assess nutritional quality.

To ensure the consumption of a healthy and nutritious diet, identifying foods that are rich in nutrients is paramount. Drewnowski developed a nutrient-rich food index that focused on consuming fewer calories but with more beneficial nutrients^(51)^. Potassium is one of the beneficial nutrients with nutrient-rich density. A study conducted in 2019 developed a nutrient-rich food score that is useful to assess the nutrient density of diets among the elderly. The tool consists of seven nutrients that should be encouraged (protein, dietary fibre, folate, vitamin D, calcium, magnesium and potassium) and three nutrients to limit (saturated fat, sodium and mono- and disaccharides)^(28)^. This 2019 study compares well with the findings of the present study which showed that potassium intake and potassium density were strongly correlated with other nutrients such as magnesium, calcium, fibre and vitamin C. Compared to the usual potassium diet, the LSHP diet showed significantly higher potassium, calcium, magnesium, fibre, vitamin C, protein and carbohydrate contents. On the other hand, the total fat and sodium content was lower.

The formulation of the LSHP diet in the present study is similar to the DASH which is high in potassium, magnesium, calcium and fibre content, and low in sodium. The DASH diet was originally proposed for the prevention and treatment of hypertension and is currently recommended as a healthy dietary standard by several countries due to its metabolic and nutritional benefits^(32–34)^. The benefits of this diet rich in fruits and vegetables are attributable to its micronutrient composition which provides increased amounts of potassium, magnesium, calcium and fibre. The diet was originally developed to contain foods that increase potassium, magnesium and calcium based on established links to lower blood pressure and successful demonstration of a clinically meaningful blood pressure-lowering effect^(32–34,52)^. Interactions between these nutrients can also have blood pressure lowering effects, such as the interaction between potassium and calcium and the ability to increase sodium excretion by the kidneys. Potassium and calcium have previously been demonstrated to interact with the renin–angiotensin system by affecting plasma renin activity, and potassium can assist with sodium balances and has also been demonstrated to potentially lower blood pressure through endothelium-dependent vascular effects^(3,53,54)^. Population studies suggested that high fruits and vegetables in DASH lower blood pressure and improve endothelial function in obese hypertensive subjects^(48)^. Moreover, the umbrella review of the systematic review concluded that the DASH dietary pattern is associated with decreased incidence of cardiovascular disease and improves blood pressure with evidence of other cardiometabolic advantages in people with and without diabetes^(34)^. In the future, the LSHP diet in the present study can be applied to increase potassium intake in healthy populations or people with NCDs.

One of the problems about a healthy diet, particularly high potassium intake, is the high cost. The present study showed that the diet cost of LSHP is significantly more expensive than the usual diet. It means that a nutrient-rich healthy diet is expensive. The daily mean cost of the LSHP diet was IDR 35 192⋅10 ± 3318⋅27 per person per day. It was categorised as high food expenditure based on daily food cost for Indonesian civil servants^(55)^. A similar categorisation was demonstrated by an epidemiological study which found that lower household expenditure and shorter education were associated with lower potassium intake^(56)^. The same trend was observed in previous studies which found that healthy diets tend to be expensive and low-cost energy-dense diets also tend to be nutrient-poor^(24–26)^. Foods that supply relatively more nutrients than calories are defined as nutrient-dense. In the present study, the LSHP diet is a more nutrient-dense and nutrient-rich food than the usual diet. It is well documented that nutrient density is an accurate marker of healthy diets, distinguishing between diets that are energy-dense and those that are nutrient-rich^(57)^. It was observed that besides potassium, the components of carbohydrate, protein, fibre, magnesium, calcium, vitamin C, fruit and potassium density were significantly higher in the LSHP diet compared to the usual diet. Adherence to healthy diet indicator (HDI) in a study in Japan is reported to be poor (6⋅6 %) and higher adherence to HDI was significantly associated with diets higher in protein, polyunsaturated fatty acids (PUFA), dietary fibre, potassium and vitamins^(23)^. The LSHP diet in the present study met the healthy diet recommendation by the WHO. It contained ≥400 g of fruits and vegetables per day, ≥25 g/d dietary fibre, ≥3500 mg/d potassium and total fat of <30 % for total energy. Furthermore, higher-quality diets were not only more costly but were also consumed by persons of higher educational levels. In contrast, foods with lower nutritional values and lower-quality diets generally cost less and tended to be consumed by groups of lower socio-economic status^(58)^. This finding is strengthened by other studies which found that nutrient density was negatively associated with energy density and positively associated with cost^(59,60)^.

The linear regression analysis in the present study showed that fibre and potassium were the most important nutrients in determining cost. This is similar to a previous study that reported a potassium-rich diet or a potassium-dense diet which was associated with higher diet costs^(15)^. In contrast, it differed from cost analysis of essential nutrients in the National Health and Nutrition Examination Survey from 2011 to 2014 that shows protein food was one of the most expensive food categories^(26)^. Interestingly, the present study proved fibre was the most nutrient costly. It might be still related to potassium since source food fibre is similar to potassium such as fruits and vegetables. Fruits and vegetables provided key nutrients at a reasonable cost when compared with other foods^(24)^. It was also be proven by other countries particularly the low-income countries where healthy foods, including fruits and vegetables, were categorised as expensive food^(59,60)^. Fruits, vegetables and complex carbohydrates together contributed to high fibre content in the menus, and they may have contributed to the high cost of fibre.

Several population studies showed that the consumption of high potassium healthy diets appears to still be a problem. There is a nutrient gap due to economic constraints in acquiring a nutritious diet. A study on diet cost in Indonesia revealed that affording a nutritious diet depended on household expenditure and geographic area. More people in the urban area can afford to consume high-quality diets^(61)^. A study by Temple and Steyn revealed that a healthy diet is unaffordable for most South Africans^(62)^. Another study showed that children from food-insecure households were more likely to consume low-quality diets, and higher parental income was associated with higher diet costs affordability^(63)^. The same trends were observed by Marty *et al.* when they found that among low-income population, actual diet cost was positively correlated with nutritional quality^(64)^. In addition, the study by Monsivais *et al.* demonstrated that higher-quality diets were costly and consumed by persons of higher educational level^(59)^. Similar findings were also reported in a previous study that a potassium-rich diet or a potassium-dense diet was associated with higher diet costs^(15)^.

The notable strength of the present study is that we identified food sources with high potassium and low sodium and utilised these directly in the formulation of healthy diet menus. Specifically, the optimal nutrients in the menus were developed based on the traditional Indonesian menu and the suitable Indonesian balance diet guideline. Therefore, the formulation of the LSHP diet was culturally acceptable, palatable and nutrient-rich. One important application of diet models is achieving a nutrient-rich diet at an affordable cost. Although we tried to arrange menus at an affordable cost, the limitation of the present study is that the LSHP diet is only applicable to the specific population and may limit generalisability to other populations. Since a large number of epidemiological studies showed that a healthy diet and nutrient-rich food are expensive, it is not easy to identify and providing healthy menus with optimal nutrients at an affordable cost is an uphill task.

## Conclusions

The main findings of the present study are that the LSHP diet had significantly higher essential nutrients than the usual diet. Potassium is one of the essential nutrients of a healthy diet, so it is important to consume the recommended high potassium healthy diet intake for maintaining health and preventing NCDs. In the present study, there was a strong positive correlation between fibre, potassium and diet cost. Since foods rich in potassium and fibre were relatively costly, cost may be a barrier to adopting a healthier diet and improving diet quality in populations. It may require a more effective and progressive strategy and price intervention from government to provide affordable and nutrient-rich food for achieving a sustainable healthy eating pattern. In addition, the menus were developed based on the traditional Indonesian menu, so they may not be applied in other ethnic groups and can not be extrapolated to a larger population. It is suggested to evaluate the menus based on different ethnicity and to a larger population to gain more comprehensive results and adaptability.

## Supplementary material

For supplementary material accompanying this paper visit https://doi.org/10.1017/jns.2021.104. click here to view supplementary material
