# Potassium intake is associated with nutritional quality and actual diet cost: a study at formulating a low sodium high potassium (LSHP) healthy diet – CORRIGENDUM

**DOI:** 10.1017/jns.2024.3

**Published:** 2024-02-08

**Authors:** Farapti Farapti, Annas Buanasita, Dominikus R. Atmaka, Stefania W. Setyaningtyas, Merryana Adriani, Purwo S. Rejeki, Yoshio Yamaoka, Muhammad Miftahussurur

The authors regret that Dr. Muhammad Miftahussurur was not listed as a corresponding author on the original publication of this paper. This has now been corrected in the article.

