# Supplementary material for: Potassium intake is associated with nutritional quality and actual diet cost: a study at formulating a low sodium high potassium (LSHP) healthy diet
Source: J Nutr Sci. 2022 Feb 16;11:e11. doi: 10.1017/jns.2021.104 (PMC8889219; doi:10.1017/jns.2021.104)
Supplement: Supplementary file 1 [file S204867902100104Xsup001.docx]

Supplemental: The content of potassium from source foods (per 100gr)

|  | 1000 kkal | 500-1000 kkal | 100-500 kkal |
| --- | --- | --- | --- |
| Potassium source foods from carbohydrate group | \|  \| \| --- \| \|  \| \|  \| \|  \| \|  \| \|  \| \|  \| \|  \| \|  \| \|  \| \|  \| \|  \| | \|  \| \| --- \| \|  \| \|  \| \|  \| \|  \| \|  \| \|  \| \|  \| \|  \| \|  \| \|  \| \|  \| \|  \| \|  \| \|  \| \|  \| \|  \| \|  \| \|  \| \|  \| \|  \| | Kasbi Sagoo, flour   \| Havermouth \| \| --- \| \| Cassava starch  Potato  Cassava \|   Half-milled rice  Biscuit  White yam  Macaroni  Boiled yam  Var rojolele, milled rice, raw  Black sticky rice, raw  Black sticky rice  Black sticky rice, mashed, raw  Sticky rice  Sticky rice, mashed, raw  Half-milled rice  Red rice, mashed, raw  Rice, flour, raw  Vermicelli  Milled yellow corn  Young corn, fresh  Potato  Pumpkin  Taro, fresh  Breadfruit, fresh  Mocaf flour  Breadfruit flour  Tapioca flour  Wheat flour |
| Potassium source foods from animal protein group | Dried shark  Jerked deer  Kaholeo  Dobo shrimp paste | Paniki with coconut milk  Awl satay  Sagoo caterpillar  Smoked cakalang  Jerked beef lung  Papay shrimp  Baronang fish  Jerked deer  Jerked crocodile  Baronang fish  Baronang fish, fresh  Snapper fish |  |
| Potassium source foods from plant protein group | Tonga bean chips  Lebui nut  Tongan bean  Red bean  Dark red bean, dried  Red bean, dried  Mung bean starch  Soy bean flour | Peanut butter  Cashewnut  Mung bean var. Bakti, dried  Mung bean, dried  Mung bean, boiled  Soy bean, dried  Soy bean, fresh  Cashewnut, fried  Cashewnut, fresh  Peanut, roasted  Peanut, boiled  Soy tempe, fried | \| String bean \| \| --- \| \| Pease \|   young pease   \| Potato cracker \| \| --- \|   Field pea  Bandanesse cannary  Tofu  Kidney bean  Boiled peanut  Roasted peanut  Dry peanut  Soymilk  Tempeh |
| Potassium source foods from fat group |  | Young coconut meat, fresh  Coconut milk | Coconut milk, with water |
| Potassium source foods from vegetables group | Sea bamboo shoot  Chinesse petai | Fern leaf  Petersely  Melinjo leaf, fresh  Papaya leaf, fresh  Black fungus, dried  Long bean, kernel, dried  Breadnut  Chinesse petai, riped, fresh | Bitter squash leaf  Lily  Watercresh  Papaya flower  Leek  Fern   \| Fern leaf \| \| --- \| \| Spinach \|   Coconut shoots  Sagoo mushroom  Cassava leaf  Pelecing kale  Seaweed  Tondano kale  Nutgrass bulbs  Moringa leaf  Long bean  Agathi flower  Celery  Field mustard  Pangium leaf  Carrot  Red/white cabbage  Lettuce   \| Nasturtium \| \| --- \|   Cucumber  Coconot shoots  Milled yellow corn  Petai  Butternut pumpkin  Bamboo shoots  Fresh bean sprout  Fresh radish  Petai  Field mustard  Seaweed  Sapodilla fruit  Eggplant  Tomato  Raw tomato  Water tomato  Angled luffa leaf  Cucumber  White flowered gourd  Chayote  White leadtree  Luffa squash  Straw mushroom  Oyster mushroom  Bean  Pease  Long bean  Spinach  Sweet leaf  Moringa leaf |
| Potassium source foods from fruits group |  | Dates palm  Balinesse banana  Bitter chocolate  Banana ketip  Durian, fresh  Dates  Riped jackfruit, fresh  Balinesse banana | \| Rotan manau  Many types of banana \| \| --- \| \| Orange  Grapes  Medan bark  Palembang pineapple  Binjai rambutan  Pineapple  Pear  Starfruit  Lime  Lemon  Many types of mango  Sweet orange  Manalagi mango  Kiwi  Peach  Red apple  Moringa fruit  Indian plum  Papaya  Duku  Balinesse banana  Avocado  Beetroot  Wild coconut  Red fruit  Manggo \|   Honeydew  Pineapple  Jackfruit  Pear  Papaya  White guava  Ambarella  Soursop  Apple  White yam  Dragon fruit  Duku  Tender coconut water  Coconut |
| Potassium source foods from dairy group | Skim milk, flour | Pregnant milk | Condensed milk   \| Goat milk \| \| --- \|   Cow’s milk  Yoghurt  Chocolate  Milk chocolate |
